# The Molecular Determinants of NEDD8 Specific Recognition by Human SENP8

**DOI:** 10.1371/journal.pone.0027742

**Published:** 2011-11-14

**Authors:** Yung-Cheng Shin, Siao-Jing Tang, Jou-Han Chen, Pei-Han Liao, Shih-Chung Chang

**Affiliations:** Department of Biochemical Science and Technology, College of Life Science, National Taiwan University, Taipei, Taiwan; National Institute for Medical Research, Medical Research Council, United Kingdom

## Abstract

Although neuronal-precursor-cell-expressed developmentally downregulated protein-8 (NEDD8) and ubiquitin share the highest level of sequence identity and structural similarity among several known ubiquitin-like proteins, their conjugation to a protein leads to distinct biological consequences. In the study, we first identified the NEDD8 protein of *Chlamydomonas reinhardtii* (CrNEDD8) and discovered that CrNEDD8 is fused at the C-terminus of a ubiquitin moiety (CrUb) in a head-to-tail arrangement. This CrUb-CrNEDD8 protein was termed CrRUB1 (related to ubiquitin 1) by analogy with a similar protein in *Arabidopsis thaliana* (AtRUB1). Since there is high sequence identity in comparison to the corresponding human proteins (97% for ubiquitin and 84% for NEDD8), a His-CrRUB1-glutathione S-transferase (GST) fusion construct was adopted as the alternative substrate to characterize the specificity of NEDD8-specific peptidase SENP8 for CrNEDD8. The data showed that SENP8 only cleaved the peptide bond beyond the di-glycine motif of CrNEDD8 and His-RUB1 was subsequently generated, confirming that SENP8 has exquisite specificity for CrNEDD8 but not CrUb. To further determine the basis of this specificity, site-directed mutagenesis at earlier reported putative molecular determinants of NEDD8 specific recognition by SENP8 was performed. We found that a single N51E mutation of CrNEDD8 completely inhibited its hydrolysis by SENP8. Conversely, a single E51N mutation of CrUb enabled this ubiquitin mutant to undergo hydrolysis by SENP8, revealing that a single residue difference at the position 51 contributes substantially to the substrate selectivity of SENP8. Moreover, the E51N/R72A double mutant of the CrUb subdomain can further increase the efficiency of cleavage by SENP8, indicating that the residue at position 72 is also important in substrate recognition. The E51N or R72A mutation of CrUb also inhibited the hydrolysis of CrUb by ubiquitin-specific peptidase USP2. However, USP2 cannot cleave the N51E/A72R double mutant of the CrNEDD8 subdomain, suggesting that USP2 requires additional recognition sites.

## Introduction

Post-translational modification by ubiquitin and ubiquitin-like proteins (Ubls) is a prominent regulatory mechanism that modulates a wide range of important cellular processes that are involved in differentiation, development, apoptosis, stress responses, the cell cycle, and the immune response [1], [2]. Ubiquitin and Ubls are initially synthesized as precursors. They must be proteolytically processed by deubiquitinating enzymes (DUBs) [3] to yield their mature forms with exposed C-terminal di-glycine motifs that covalently conjugate to lysine residues of target proteins via isopeptide-bond linkages [4]. This conjugation process is catalyzed sequentially by E1 activating enzymes, E2 conjugating enzymes and, in several cases, E3 ligases that recognize target proteins or facilitate Ubl transfer from an E2 to a target. The cycle can also be reset using a procedure that is called deconjugation by removing Ubls from targets by DUBs [5].

Various Ubls that share sequence similarity with ubiquitin have been identified. In particular, the neural-precursor-cell-expressed developmentally downregulated protein-8 (NEDD8) in humans [6], or related to ubiquitin 1 (RUB1) in yeast and *Arabidopsis* [7], [8], is the closest relative to ubiquitin and can be conjugated to substrates in a process that is similar to ubiquitination, called neddylation. Despite its high degree of sequence identity (∼60%) and structural similarity [9] with ubiquitin, NEDD8 depends on its dedicated E1 enzyme, a heterodimer that consists of the amyloid precursor protein-binding protein (APP-BP1) and the Uba3 protein, as well as the E2 enzyme (Ubc12) for conjugation to cellular targets [10], [11], [12].

Although many proteases exhibit dual specificity for ubiquitin and NEDD8 precursors, including USP21 [13], Ataxin-3 [14], PfUCH54 [15] and UCH-L3 in humans [16], [17] or Yuh1 in *Saccharomyces cerevisiae* [18], a group of DUBs exhibits a strong ability to catalyze NEDD8 precursor processing and NEDD8 deconjugation (also known as deneddylation) from neddylated substrates. A cysteine protease with unique specificity for NEDD8 has been identified in human cells. Human deneddylase 1 (DEN1) [19], [20], also called NEDD8-specific protease (NEDP1) [21], is a member of the ULP/SENP peptidase family and was initially designated as SENP8 (SUMO-1/sentrin/SMT3-specific peptidase 8) [22]. DEN1 is highly conserved throughout evolution and members of its family can be found in *Schizosaccharomyces pombe* [23], *Drosophila* [24], *Arabidopsis* and mammals [21]. DEN1/NEDP1 has a 60,000-fold preference for NEDD8 over ubiquitin [19] and cannot cleave ubiquitin or small ubiquitin-like modifier (SUMO) precursors with C-terminal extensions [21]. However, it exhibits remarkable proteolytic activity against NEDD8 precursor and NEDD8-conjugated cullin proteins (CULs) *in vitro* and *in vivo* [20], [24]. DEN1 has been suggested to be more efficient at deneddylating hyper-neddylated CUL1 but less efficient at removing NEDD8 from mono-neddylated CUL1 [20]. Apart from DEN1, the best-characterized NEDD8 isopeptidase is the CSN-5 subunit of the COP9 signalosome (CSN), which contains a Jab1/MPN domain metalloenzyme (JAMM) motif and can deneddylate CULs [25], [26]. Notably, unlike DEN1, CSN can deneddylate mononeddylated CUL1 under physiological conditions, but does not participate efficiently in the deconjugation of hyper-neddylated CUL1 [20].

Since ubiquitin and NEDD8 modifications have distinct biological consequences, a highly specific mechanism that allows cells to discriminate between ubiquitin and NEDD8 must exist. This important fidelity is established by specific enzyme systems that correctly catalyze the conjugation and deconjugation of each of these two modifiers to match their corresponding cascades. Notably, a conserved basic residue in Uba3 acts as a selectivity gate by repelling the Arg-72 in ubiquitin but not the corresponding alanine residue in NEDD8 to prevent misactivation of ubiquitin by the NEDD8 E1 [27], [28]. Conversely, Arg-72 has been shown to play a key role in the preferential activation of ubiquitin over NEDD8 by ubiquitin E1 [9], [29]. Additionally, Ubc12 exhibits a vestigial preference for ubiquitin over NEDD8 and has unique surface elements that inhibit its interaction with ubiquitin E1 but promote its interaction with NEDD8 E1 to form a thioester-linked Ubc12-NEDD8 product [30], [31]. Similarly, NEDD8-specific protease must have the capability to discriminate between ubiquitin and NEDD8. More evidence of the basis of NEDD8 selectivity determinants was obtained in a study of the structure of NEDD8 bound to DEN1/NEDP1 [32], [33]. The structure reveals that NEDP1 undergoes a drastic conformational change when it binds to NEDD8. Structural, mutational and biochemical analyses have revealed key residues that participate in molecular recognition and elucidated how a single-residue change between NEDD8 and ubiquitin is significantly involved in discrimination by NEDP1 [33]. The results demonstrate that Ala-72 in NEDD8 (Arg-72 in ubiquitin) is an important—but not the sole—determinant of NEDP1 selectivity for NEDD8 over ubiquitin [32], [33].

The protein deneddylation system of *Chlamydomonas reinhardtii* has not been investigated. The present study focused on the identification and characterization of *C. reinhardtii* NEDD8 (CrNEDD8). Interestingly, in many plants, NEDD8 is expressed as the C-terminal part of a bi-ubiquitin fusion protein, which comprises an N-terminal ubiquitin and a C-terminal NEDD8 in a head-to-tail arrangement. Since the bi-ubiquitin of *Arabidopsis thaliana* has been designated AtRUB1 (related to ubiquitin 1), the bi-ubiquitin of *C. reinhardtii* is herein named CrRUB1. Regarding the unique arrangement of CrRUB1 sequence and the high sequence identity in comparison to the corresponding human proteins, CrRUB1 was considered to be a suitable substrate for investigating the catalytic specificity of SENP8. The results of site-directed mutagenesis and biochemical analyses demonstrated that SENP8 specifically discriminated between CrUb and CrNEDD8 by recognizing the corresponding residues at positions 51 and 72 in CrUb and CrNEDD8. The residue Asn-51 in CrNEDD8 is critical for substrate recognition by SENP8. Mutation of CrNEDD8 Asn-51 to the corresponding residue Glu-51 in CrUb completely suppressed the hydrolysis of the C-terminal di-glycine motif of CrNEDD8 by SENP8. However, a single mutation of CrUb Glu-51 with Asn-51 in CrNEDD8 made this CrUb mutant capable of undergoing hydrolysis by SENP8. Additionally, the E51N/R72A double mutant of the CrUb subdomain can further promote its hydrolysis by SENP8, indicating that position 72 is also important in substrate recognition. Notably, the residues at positions 51 and 72 in ubiquitin and NEDD8, respectively, are highly conserved among different organisms, suggesting that these two key determinants may play important roles in NEDD8 specific recognition by SENP8.

## Results

### Identification of the bi-ubiquitin gene from *C. reinhardtii*

**Figure 1:**
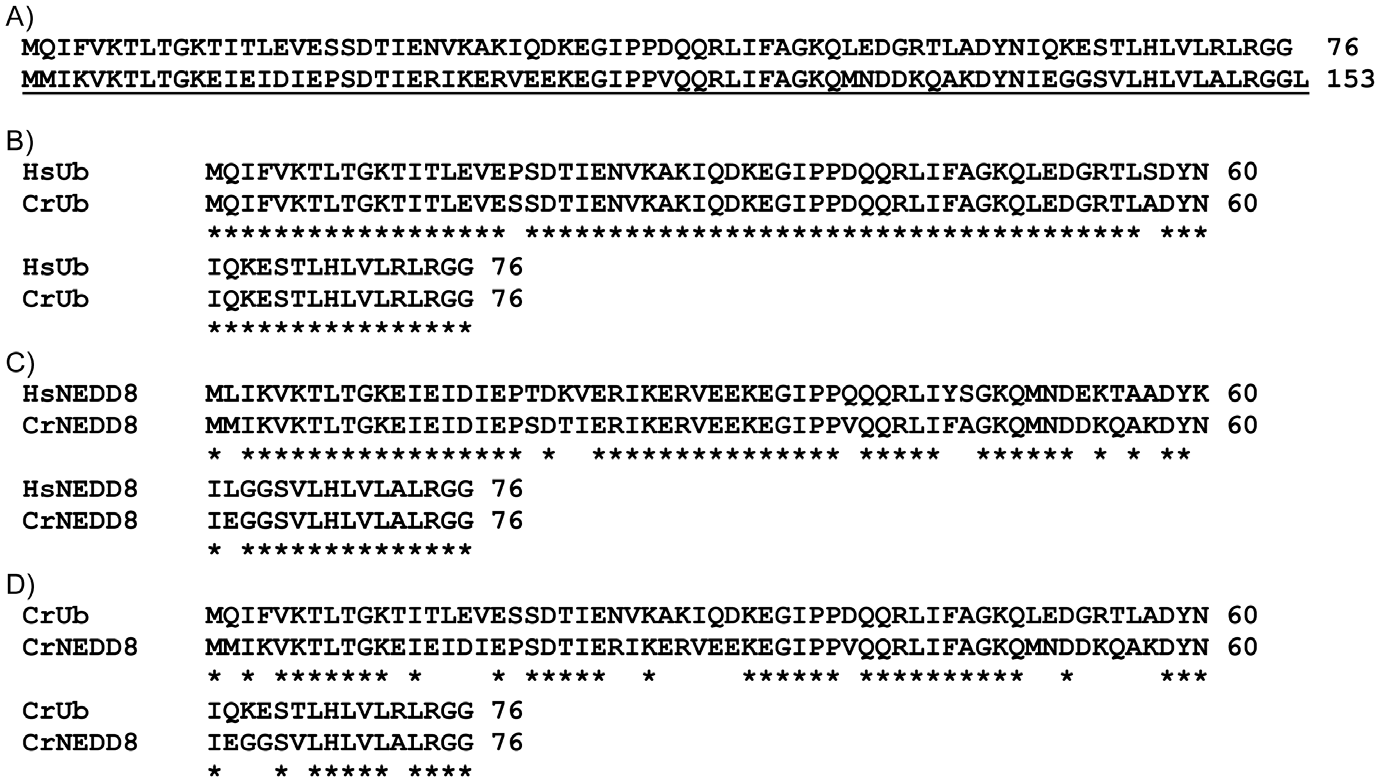
Sequence alignments of CrRUB1 with human ubiquitin and NEDD8. (**A**) The amino acid sequence of CrRUB1. CrRUB1 is composed of an N-terminal ubiquitin (CrUb) and a C-terminal NEDD8 (CrNEDD8) in a head-to-tail arrangement. The sequence of CrNEDD8 from residue 77 to residue 153 in CrRUB1 was underlined. (**B**) Sequence comparison of CrUb with human ubiquitin (HsUb). (**C**) Sequence comparison of CrNEDD8 with human NEDD8 (HsNEDD8). (**D**) Sequence comparison of CrUb with CrNEDD8. Sequences were aligned using ClustalW. Identical residues were marked with asterisks.

Evidently, NEDD8 is highly conserved in most eukaryotes [34], [35], [36]. To identify the NEDD8 protein of *C. reinhardtii*, a protein BLAST alignment search was initially conducted using a human NEDD8 amino acid sequence as the query over *C. reinhardtii* v4.0 genomic scaffolds by the Department of Energy Joint Genome Institute (JGI). A 153-residual protein was identified. Interestingly, this *C. reinhardtii* protein exists as a bi-ubiquitin (named CrRUB1) that comprises an N-terminal ubiquitin (named CrUb) and a C-terminal NEDD8 (named CrNEDD8) in a head-to-tail arrangement (Fig. 1A). Indeed, CrUb shares 97% identity with human ubiquitin (Fig. 1B) and CrNEDD8 shares 84% identity with human NEDD8 (Fig. 1C). The alignment of the sequences of CrUb and CrNEDD8 showed that they share 63% identity (Fig. 1D).

**Figure 2:**
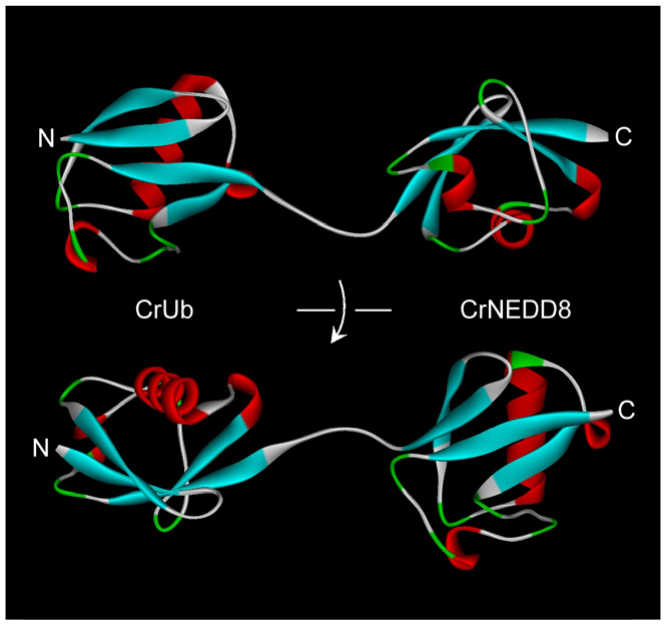
The predicted polypeptide folds of CrRUB1. The presumed 3-D structure of CrRUB1 was simulated by the Discovery Studio® protein modeling program using the crystal structure of linear di-ubiquitin (PDB ID code 2W9N) as the template. The secondary structures of β-sheet, α-helix and coil were shown in cyan, red and green, respectively. The letters “N” and “C” indicate the N terminus and the C terminus of CrRUB1, respectively.

Because of the high sequence similarity and the presumed structural correlation among various ubiquitin and NEDD8 homologues, the structure of CrRUB1 was predicted by simulation using the Discovery Studio® (Accelrys, Inc.) protein modeling program with the available crystal structure of linear di-ubiquitin (PDB ID code 2W9N [37]) as the reference template. The polypeptide folds highlight that CrUb and CrNEDD8 have similar structural features, including four β-sheets and one major α-helix (Fig. 2). Clearly, distinguishing CrUb from CrNEDD8 by comparing their main structural features is difficult. Therefore, some local non-conserved residues in CrUb and CrNEDD8 are predicted to be the molecular determinants of substrate discrimination that is recognized by ubiquitin-specific peptidase or NEDD8-specific peptidase.

### SENP8 exhibits very high specificity for CrNEDD8 but not CrUb

**Figure 3:**
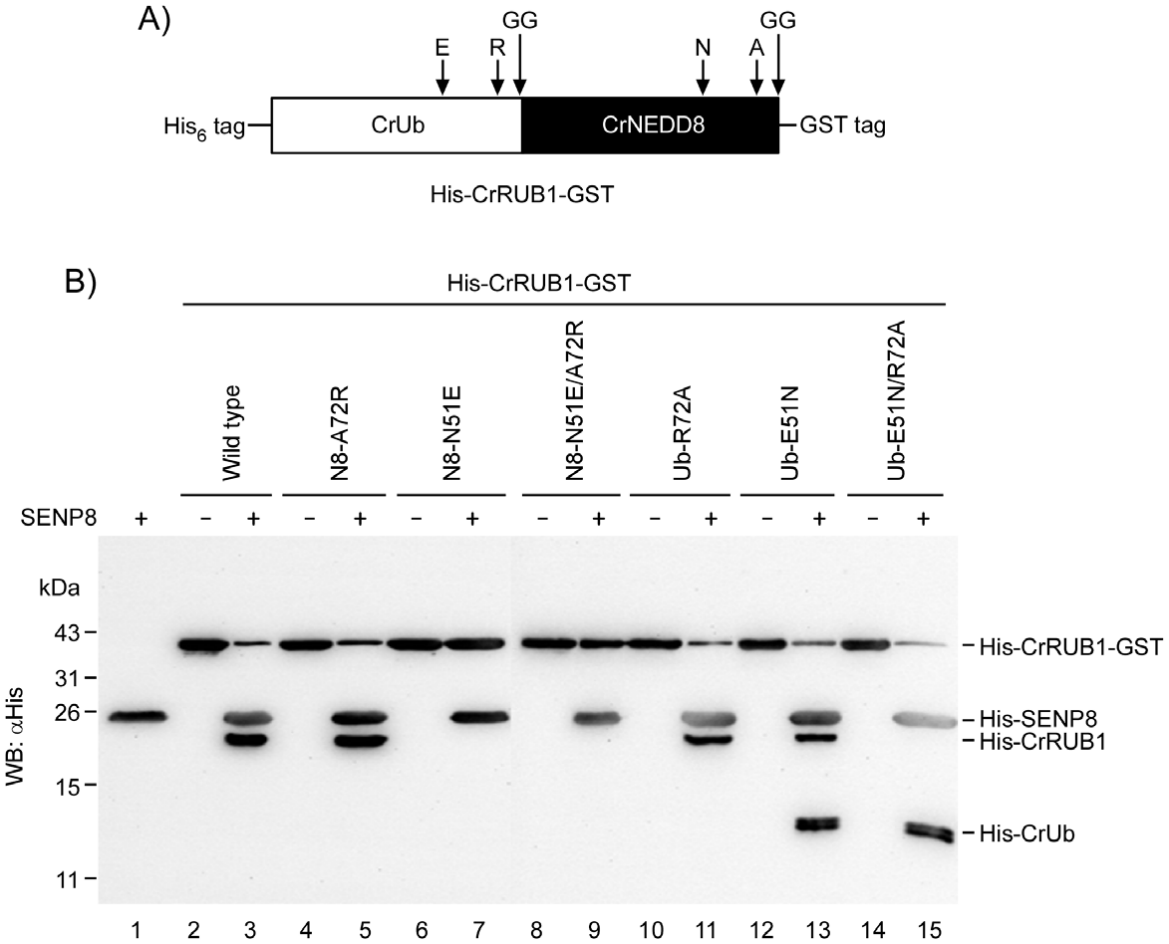
Discrimination of CrNEDD8 and CrUb by SENP8 is through recognition of the residues at positions 51 and 72. (**A**) The schematic diagram indicating the key residues involved in NEDD8 ubiquitin discrimination by SENP8. The letters “E” and “R” indicate Glu-51 and Arg-72 in CrUb, and “N” and “A” indicate Asn-51 and Ala-72 in CrNEDD8, respectively. The di-glycine motif is noted as “GG”. The recombinant CrRUB1 substrate used in the study comprises an N-terminal (His)_×6_ tag and a C-terminal GST-fusion protein. (**B**) 1.5 µg of His-CrRUB1-GST and indicated mutants were incubated with or without 2.4 µg of SENP8 at 37°C for two hours. All reactions were terminated by incubating with 4× SDS-PAGE sample buffer at 100°C for 10 min. Samples were separated on 16.6% SDS-PAGE and further analyzed using western blotting with the anti-(His)_×6_ tag antibody. N8 indicates CrNEDD8 in the figure.

To determine whether NEDD8-specific peptidase SENP8 is specific for CrNEDD8 but not CrUb, the His-CrRUB1-glutathione S-transferase (GST) fusion protein (Fig. 3A) was prepared with a GST tag fused at the C-terminus of the di-glycine motif of CrNEDD8 for incubation with SENP8 to verify its catalytic selectivity. If SENP8 can only cleave at the peptide bond beyond the di-glycine motif of CrNEDD8, then the His-CrRUB1 and a free GST are produced. Western blotting data demonstrated that the amount of His-CrRUB1-GST considerably declined upon incubation with SENP8, and much His-CrRUB1 was produced (Fig. 3B, *lane* 3). Since CrUb was not detected in the experiment, SENP8 exclusively cleaved at the peptide bond beyond the di-glycine motif of CrNEDD8, revealing that it exhibits a very high substrate specificity toward CrNEDD8 but not toward CrUb.

### Molecular determinants of CrNEDD8 CrUb discrimination by SENP8

Many of the residues in NEDD8 that interact with NEDP1/DEN1/SENP8 are conserved in ubiquitin and therefore cannot be the determinants that are involved in discrimination between NEDD8 and ubiquitin. Position 72 which differs between ubiquitin and NEDD8 (Fig. 1C) has been assumed to be a residue that can be used by SENP8 in discriminating between ubiquitin and NEDD8, as demonstrated in the case of NEDD8 E1-activating enzyme complex [27], [28]. To examine whether position 72 in CrUb or CrNEDD8 is also involved in substrate recognition by SENP8, the susceptibility of wild-type CrRUB1, CrRUB1_NEDD8(A72R) mutant and CrRUB1_Ub(R72A) mutant to cleavage by SENP8 was evaluated. Surprisingly, SENP8 exhibited similar efficiencies in degrading wild-type CrRUB1, CrRUB1_NEDD8(A72R) and CrRUB1_Ub(R72A) (Fig. 3B, *lane* 3, *lane* 5 & *lane* 11), indicating that position 72 is not the major determinant of substrate discrimination by SENP8. Another residue which has been found not equally well conserved in ubiquitin or NEDD8 is position 53, where the residue is Gly or Arg in ubiquitin and the equivalent residue is Asp or Glu in NEDD8 respectively [9], [33]. Although, it has been reported that the residue at position 53 in NEDD8 appeard to have only limited potential to make direct contacts with SENP8 [33], the CrRUB1_Ub(G53D) mutant and the CrRUB1_NEDD8(D53G) mutant were still prepared to test whether the residue at position 53 can be the critical site for NEDD8 specific recognition by SENP8. We found that SENP8 also exhibited similar efficiencies in degrading wild-type CrRUB1, CrRUB1_Ub(G53D) mutant and CrRUB1_NEDD8(D53G) mutant, and the entire CrRUB1 fragments were markedly observed in the experiments (Fig. S1). The data clearly demonstrated that mutation of the corresponding residue at position 53 in CrUb or CrNEDD8 did not affect the catalytic activity and substrate specificity of SENP8. Based on the above observations, the discrimination of CrUb and CrNEDD8 by SENP8 is more complex than has been predicted.

Another sequence feature of ubiquitin and NEDD8 has been noted. Asn-51 is unique to NEDD8 because the equivalent position is substituted to glutamate in ubiquitin and glutamine in SMT3 [6], [38]. In an earlier investigation, Reverter et al. found that E51N ubiquitin mutant only weakly inhibited the DEN1-catalyzed hydrolysis of ubiquitin-7-amido-4-methyl coumarin, suggesting that position 51 may be insubstantial to this phenomenon [32]. However, the result in the present study revealed that the mutation of CrNEDD8 Asn-51 to the corresponding residue Glu-51 in CrUb completely inhibited its hydrolysis by SENP8 (Fig. 3B, *lane* 7). Furthermore, a complete inhibition of the hydrolysis by SENP8 was also observed using CrRUB1_NEDD8(N51E/A72R) double mutant as the substrate (Fig. 3B, *lane* 9). To verify that position 51 indeed contributed to the ability of SENP8 to recognize the substrate, the equivalent mutant CrRUB1_Ub(E51N) was utilized. The data thus obtained that the single E51N mutation of CrUb may make CrRUB1_Ub(E51N) able to undergo hydrolysis by SENP8 at the C-terminal di-glycine motif of CrUb, because both His-CrRUB1 and His-CrUb were produced when His-CrRUB1-GST was hydrolyzed (Fig. 3B, *lane* 13). This finding provides evidence that position 51 is a molecular determinant of CrNEDD8 CrUb discrimination by SENP8. Moreover, the E51N/R72A double mutant of the CrUb subdomain further increased the efficiency of cleavage of CrRUB1_Ub(E51N/R72A) by SENP8 to produce His-CrRUB1 and His-CrUb (Fig. 3B, *lane* 15), indicating that position 72 was importantly involved in substrate recognition.

**Figure 4:**
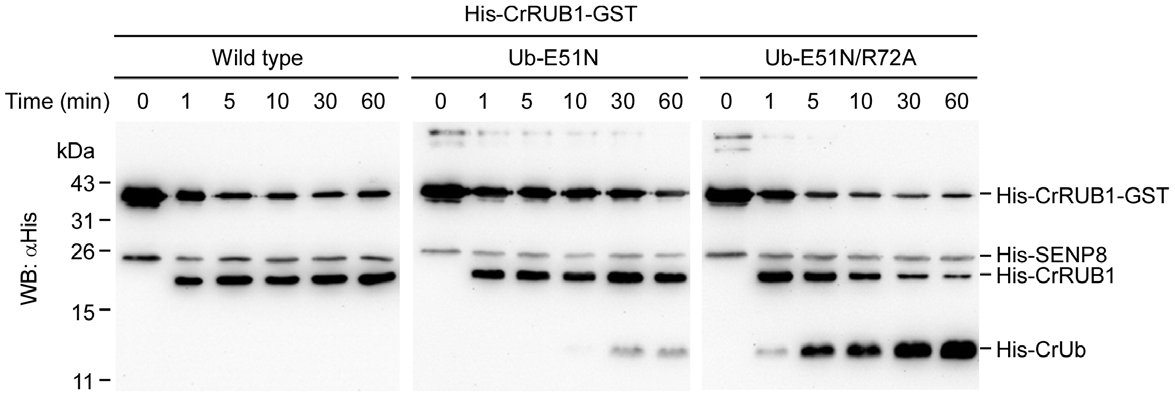
Comparison of the degradation rates of wild-type CrRUB1, CrRUB1_Ub(E51N) and CrRUB1_Ub(E51N/R72A) catalyzed by SENP8. In the time course experiment, 1.5 µg of His-CrRUB1-GST, His-CrRUB1(Ub-E51N)-GST mutant or His-CrRUB1(Ub-E51N/R72A)-GST mutant was incubated with 2.4 µg of SENP8 at 37°C for indicated time points. All reactions were terminated by incubating with 4× SDS-PAGE sample buffer at 100°C for 10 min, and samples were separated on 16.6% SDS-PAGE and then analyzed by western blotting with the anti-(His)_×6_ tag antibody.

To compare the rates of degradation of wild-type CrRUB1, CrRUB1_Ub(E51N) and CrRUB1_Ub(E51N/R72A) which were catalyzed by SENP8, a time course experiment was performed. As described above, since the di-glycine motif of CrNEDD8 in CrRUB1 was the sole cleavage site of SENP8, considerable His-CrRUB1 was formed as the amount of His-CrRUB1-GST substrate declined (Fig. 4, left panel). When His-CrRUB1_Ub(E51N)-GST was incubated with SENP8 for various periods, His-CrRUB1 was efficiently produced within the first minute, whereas His-CrUb was not detected for 10 min (Fig. 4, middle panel), revealing that SENP8 still preferentially cleaved CrNEDD8 rather than CrUb(E51N). In other words, although SENP8 can cleave CrUb(E51N), CrUb(E51N) remains less accessible than CrNEDD8 for cleavage. However, when CrRUB1_Ub(E51N/R72A) was used as the substrate, both His-RUB1 and His-CrUb were produced within the first minute and CrRUB1 was further cleaved to obtain more CrUb as the incubation time increased (Fig. 4, right panel). Comparing the amount of CrUb that was formed by SENP8 using CrRUB1_Ub(E51N) as the substrate with that formed using CrRUB1_Ub(E51N/R72A) as the substrate clearly demonstrated that the additional R72A mutation in combination with the E51N mutation greatly promoted the hydrolysis of the CrUb mutant.

### USP2 cannot cleave CrNEDD8 with A72R and N51E mutations

**Figure 5:**
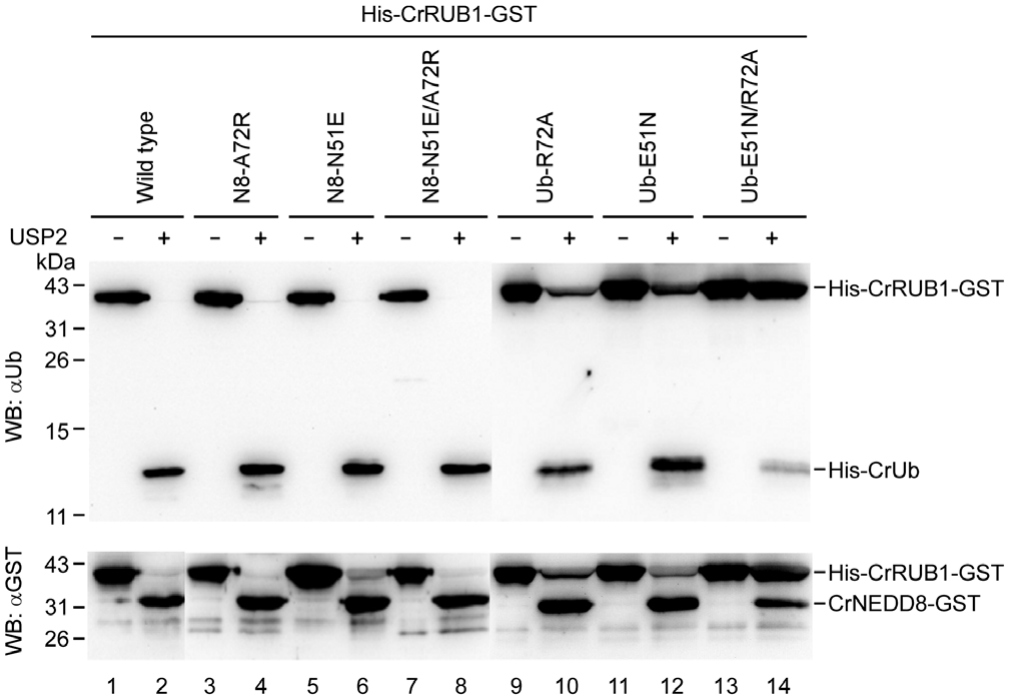
Activities of USP2 on processing of CrNEDD8 and CrUb with mutations at positions 72 and 51. His-CrRUB1-GST and indicated mutants (1.5 µg) were incubated with or without 2 µg of USP2 at 37°C for two hours. All reactions were terminated by incubating with 4× SDS-PAGE sample buffer at 100°C for 10 min. Samples were separated on 16.6% SDS-PAGE and further analyzed using western blotting with the anti-ubiquitin antibody (upper panel) and the anti-GST antibody (lower panel). N8 indicates CrNEDD8 in the figure.

To determine whether positions 51 and 72 also contribute to the discrimination of CrNEDD8 and CrUb by ubiquitin-specific peptidase USP2, the same sets of substrates as were used in Fig. 4 were incubated with USP2. Regardless of whether the substrate was wild-type CrRUB1, CrRUB1_NEDD8(A72R), CrRUB1_NEDD8(N51E) or CrRUB1_NEDD8(N51E/A72R), USP2 could not hydrolyze the C-terminal di-glycine motif of CrNEDD8 because only His-CrUb and CrNEDD8-GST were observed in the experiments when these substrates were cleaved (Fig. 5, *lane* 2, *lane* 4, *lane* 6 & *lane* 8). The results suggested that position(s) in CrNEDD8 other than 51 and 72 have entities that can strongly inhibit CrNEDD8's availability for hydrolysis by USP2.

### Mutation of residue at position 51 or position 72 in CrUb inhibits its hydrolysis by USP2

The mutation of ubiquitin Arg-72 to Ala-72 can markedly inhibit its hydrolysis by ubiquitin-specific peptidase HAUSP [33]. This investigation sought to determine whether USP2 exhibits a substrate discrimination mechanism which is similar to that of HAUSP. The data showed that USP2 cleaved CrUb-R72A less efficiently than it cleaved wild-type CrUb (Fig. 5, *lane* 10), revealing that position 72 in ubiquitin may be a common molecular determinant of substrate recognition by HAUSP and USP2. CrUb with an E51N mutation was also cleaved less efficiently than wild-type CrUb by USP2 (Fig. 5, *lane* 12). Additionally, the E51N/R72A double mutant of the CrUb subdomain more significantly inhibited the degradation of the substrate and reduced the amounts of His-CrUb and CrNEDD8-GST which were produced by USP2 (Fig. 5, *lane* 14), suggesting that positions 51 and 72 in CrUb were the critical molecular determinants of substrate recognition by USP2.

## Discussion

In this study, a bi-ubiquitin protein from *C. reinhardtii*, named CrRUB1, was initially identified. Analysis of its sequence revealed that it comprised an N-terminal ubiquitin (CrUb) and a C-terminal NEDD8 (CrNEDD8) in a head-to-tail arrangement (Fig. 1). Notably, the ubiquitin-NEDD8 fusion protein was found in many plant organisms but not in animals (Fig. S2). The predicted structures of CrUb and CrNEDD8 were very similar (Fig. 2). Because of the unique arrangement of CrRUB1 sequence and the high sequence identity in comparison to the corresponding human proteins (97% for ubiquitin and 84% for NEDD8), this naturally occurring ubiquitin-NEDD8 fusion protein was regarded as an ideal substrate for investigating the specific mechanism by which SENP8 recognizes CrUb and CrNEDD8. In the present study, a His-CrRUB1-GST fusion construct was adopted as the alternative substrate to assay the catalytic activity of SENP8 through detecting the removal of the C-terminal GST tag and the generation of His-CrRUB1 by western blotting. SENP8 will digest the peptide bond between the di-glycine motif of CrNEDD8 and the C-terminal GST tag. In earlier studies, Reverter et al. found that ubiquitin with the R72A or E51N mutation only weakly inhibited the DEN1-catalyzed hydrolysis of ubiquitin-7-amido-4-methyl coumarin [32], revealing that position 72 or 51 was not the sole determinant of NEDD8 ubiquitin discrimination by DEN1. However, Shen et al. showed that NEDD8 A72R mutant was cleaved significantly more slowly by NEDP1 than was the wild-type NEDD8 and ubiquitin R72A mutant increased the efficiency of cleavage by NEDP1, suggesting that position 72 was an important determinant [33]. Therefore, the role of position 72 in the discrimination between ubiquitin and NEDD8 by NEDP1/DEN1 remains controversial. The results of the experiments in this study demonstrated that the mutation of CrNEDD8 Ala-72 to the corresponding residue Arg-72 in CrUb did not influence hydrolysis by SENP8, whereas the mutation of CrNEDD8 Asn-51 to the corresponding residue Glu-51 in CrUb completely inhibited the hydrolysis (Fig. 3B, *lane* 7). In contrast, the E51N mutation of CrUb enabled this ubiquitin mutant to undergo hydrolysis by SENP8 (Fig. 3B, *lane* 13 and Fig. 4, middle panel). Additionally, the E51N/R72A double mutant of the CrUb subdomain can further promote hydrolysis by SENP8 (Fig. 3B, *lane* 15 and Fig. 4, right panel). The data revealed that both residues at positions 51 and 72 served as molecular determinants of CrNEDD8 specific recognition by SENP8. Position 51 is likely to be a critical determinant while position 72 is a helper. It is noteworthy to mention that NEDD8 E1 also determines the substrate by a similar mechanism, which involves recognition of the residue at position 72 between ubiquitin and NEDD8 [27], [28].

Structural analyses of a complex between NEDD8 and DEN1/NEDP1 suggested that position 72 might contribute importantly to the selectivity of DEN1/NEDP1 for NEDD8 over ubiquitin [32], [33]. Based on the inability of ubiquitin_R72A mutant to inhibit the hydrolysis of ubiquitin-7-amido-4-methylcoumarin by DEN1/NEDP1 [32] and the synthetic bi-ubiquitin substrate (His-MBP-NEDD8(m)-Ub) containing NEDD8_A72R mutant was poorly digested by DEN1/NEDP1 [33], the established studies obtained opposite results by using different experimental methods. In the present study, a novel bi-ubiquitin substrate from *C. reinhardii* containing an N-terminal ubiquitin moiety and a C-terminal NEDD8 subdomain is similar with the synthetic bi-ubiquitin substrate. Thus, consistent with the suggestion as suggested by Shen et al., we posit that differences between interpretations of experimental results may arise from conducting of different biochemical analyses. However, this study provided more evidence of the function of position 51 in ubiquitin and NEDD8, and demonstrated its importance in the recognition of the substrate by SENP8.

Experiments to determine whether positions 51 and 72 contributed to the discrimination between CrNEDD8 and CrUb by USP2 revealed that USP2 could not cleave CrNEDD8 mutants when the corresponding residues of positions 51 and 72 in CrNEDD8 were substituted with those in CrUb (Fig. 5.) The data suggested that USP2 may use multiple determinants to prevent inappropriate degradation of CrNEDD8, and the residues that contributed to its substrate selectivity should be located in other non-conserved sequences between CrUb and CrNEDD8. However, USP2 cleaved CrUb with the E51N or R72A mutation at a reduced efficiency (Fig. 5), revealing that both of positions 51 and 72 were involved in the interaction with USP2. Therefore, based on the biochemical analysis of CrRUB1 with various ubiquitin-like protein peptidases, SENP8 and USP2 were determined possibly to use different molecular mechanisms to distinguish NEDD8 from ubiquitin or vice versa.

## Materials and Methods

### *Chlamydomonas* strain and culture condition

The strain number and culture condition of *C. reinhardtii* cells were described previously [39]. Briefly, *C. reinhardtii* cells were cultured at 25°C in the AC medium containing 10 mM NaNO_3_, 1 mM MgSO_4_, 0.1 mM CaCl_2_, 0.2 mM KH_2_PO_4_ and 1.2 mM K_2_HPO_4_ with sufficient air supply and under continuous lighting. For RNA isolation, cells were cultured to a density of 6–7×10^7^ cells/mL.

### Total mRNA isolation and reverse transcription

Total mRNA was isolated from *C. reinhardtii* cells using FastTrack 2.0 mRNA Isolation Kit (Invitrogen, Carsbad, CA) according to the manufacturer's instruction. The reverse transcription of the first-strand cDNA was performed at 37°C for 50 min using M-MLV (Moloney Murine Leukemia Virus) Reverse Transcriptase (Invitrogen, Carsbad, CA) according to the manufacturer's instruction.

### Plasmid construction

The gene encoding for *C. reinhardtii* bi-ubiquitin (CrRUB1) was identified by Basic Local Alignment Search Tool (BLAST) using human NEDD8 gene sequence as query on the JGI (genome.jgi-psf.org/chlre4) and NCBI (www.ncbi.nlm.nih.gov) *C. reinhardtii* genome databases. Multiple sequence alignments of CrRUB1, human ubiquitin and human NEDD8 were analyzed by the ClustalW algorithm (www.ebi.ac.uk/Tools/clustalw). The nucleotide sequence data of CrRUB1 reported will appear in Genbank®, EMBL, DDBJ and GSDB Nucleotide Sequence Databases under the accession number HM629426.

The cDNA encoding for full-length CrRUB1 was amplified by standard PCR method using Phusion High-Fidelity PCR Kit (Finnzymes Oy, Espoo, Finland) with the following primer set: CrRUB1-forward, 5′-GCGGATCCATGCAGATTTTCGTCAAGAC-3′ (BamHI site underlined) and CrRUB1-reverse, 5′-GCCTCGAGTCAGAGCCCGCCACGCAGAG-3′ (XhoI site underlined). For constructing the His-CrRUB1-GG-GST expression vector, the cDNA of CrRUB1-GG flanked with BamHI and XhoI was cloned into the pET-28a plasmid (Novagen, EMD Biosciences, San Diego) without a stop codon. The GST coding region was then introduced into the same plasmid with the correct reading frame using pGEX-4T-1 as the PCR template with the forward primer 5′-GGCTCGAGATGTCCCCTATACTAGGTTATTGG-3′ (XhoI site underlined), and the reverse primer 5′-CTGCTCAGCTCAATCCGATTTTGGAGGATGGTCG-3′ (BlpI site underlined). The sequence of the His-CrRUB1-GG-GST expression vector was further verified by DNA sequencing.

### Site-directed mutagenesis

All CrRUB1 mutants were generated by PCR-based QuikChange™ Site-Directed Mutagenesis Kit (Stratagene, La Jolla, CA), according to the manufacturer's instruction. The primers used for the mutagenesis were shown in Table S1. All mutations were confirmed by DNA sequencing.

### Protein expression and purification

Expression vectors containing His-CrRUB1-GST, CrRUB1_Ub(R72A), CrRUB1_Ub(E51N), CrRUB1_Ub(G53D), CrRUB1_Ub(E51N/R72A), CrRUB1_NEDD8(A72R), CrRUB1_NEDD8(N51E), CrRUB1_N8(D53G), CrRUB1_NEDD8(A72R/N51E), His-SENP8 (provided by Guy Salvesen, Burnham Institute for Medical Research, La Jolla, CA) and His-USP2-core (provided by Daniel Taillandier, McGill Universiry, Canada) were transformed into *E. coli* BL21(DE3) cells (Novagen, EMD Biosciences, San Diego) individually. Cells were incubated at 37°C on an orbital shaker at 150 rpm. Expression of the recombinant protein was induced at an A_600_ of 0.6–0.7 by adding isopropyl-1-thio-β-D-galactopyranoside to a final concentration of 0.5 mM for three hours. His-tagged proteins were purified using HisTrap™ FF column (GE Healthcare, Piscataway, NJ) and bound proteins were eluted with a 20–500 mM gradient of imidazole in 20 mM NaH_2_PO_4_, pH 7.4 and 500 mM NaCl. GST-tagged proteins were purified using GSTrap™ FF column (GE Healthcare, Piscataway, NJ) and bound proteins were eluted with 20 mM reduced glutathione in 50 mM Tris-HCl, pH 8.0. The protein purity was examined by 16.6% SDS-PAGE and the concentration was determined by the Bradford dye-binding method [40].

### Peptidase activity assay

To measure the peptidase activity, 1.5 µg of purified His-CrRUB1-GST was incubated with 2.4 µg of purified recombinant SENP8 or 2 µg of purified recombinant USP2-core at 37°C in buffer containing 50 mM Tris-HCl, pH 8.0, 50 mM NaCl and 5 mM β-mercaptoethanol. All reactions were terminated by incubating with 4× SDS-PAGE sample buffer at 100°C for 10 min. Samples were separated on 16.6% SDS-PAGE and further analyzed by western blotting using the anti-His antibody (GE Healthcare, Piscataway, NJ), anti-ubiquitin antibody (Sigma-Aldrich Co.) or anti-GST antibody (Santa Cruz Biotechnology, Inc.).

## Supporting Information

Figure S1 **The residue at position 53 is not a crucial site for NEDD8 specific recognition by SENP8.** 1.5 µg of His-CrRUB1-GST and indicated mutants at position 53 of CrUb and CrNEDD8 were incubated with 2.4 µg of SENP8 or 2 µg of USP2 at 37°C for two hours. All reactions were terminated by incubating with 4× SDS-PAGE sample buffer at 100°C for 10 min. Samples were separated on 16.6% SDS-PAGE and further analyzed using western blotting with the anti-(His)_×6_ tag antibody. N8 indicates CrNEDD8 in the figure.
(DOC)

Figure S2 **Sequence alignment of NEDD8 proteins from different plant organisms.** Residues of position 51 and position 72 are indicated in square boxes respectively. As noted, ubiquitin contains a negatively charged residue at position 51, whereas NEDD8 contains a corresponding non-polar residue. Position 72 in ubiquitin is an arginine but not the corresponding alanine residue in NEDD8. Cr, *Chlamydomonas reinhardtii*; Vc, *Volvox carteri*; Ps, *Picea sitchensis*; Pp, *Physcomitrella patens*; Os, *Oryza sativa*; Sb, *Sorghum bicolor*; At, *Arabidopsis thaliana*; Da, *Deschampsia antarctica*; Zm, *Zea mays*; Gm, *Glycine max*; Vv, *Vitis vinifera*; Rc, *Ricinus communis*; Pt, *Populus trichocarpa*.
(DOC)

Table S1 **The list of the primers used for the mutagenesis in the study.** All CrRUB1 mutants were generated by PCR-based QuikChangeTM Site-Directed Mutagenesis Kit (Stratagene, La Jolla, CA), according to the manufacturer's instruction. The primers used for the mutagenesis were listed in the table. N8 indicates CrNEDD8.
(DOC)
