# Supplementary material for: The Molecular Determinants of NEDD8 Specific Recognition by Human SENP8
Source: PLoS One. 2011 Nov 14;6(11):e27742. doi: 10.1371/journal.pone.0027742 (PMC3215745; doi:10.1371/journal.pone.0027742)
Supplement: Figure S1 — The residue at position 53 is not a crucial site for NEDD8 specific recognition by SENP8. 1.5 µg of His-CrRUB1-GST and indicated mutants at position 53 of CrUb and CrNEDD8 were incubated with 2.4 µg of SENP8 or 2 µg of USP2 at 37°C for two hours. All reactions were terminated by incubating with 4× SDS-PAGE sample buffer at 100°C for 10 min. Samples were separated on 16.6% SDS-PAGE and further analyzed using western blotting with the anti-(His)×6 tag antibody. N8 indicates CrNEDD8 in the figure. (DOC) [file pone.0027742.s001.doc]

**SUPPORTING INFORMATION**

**Figure S1 The residue at position 53 is not a crucial site for NEDD8 specific recognition by SENP8.** 1.5 μg of His-CrRUB1-GST and indicated mutants at position 53 of CrUb and CrNEDD8 were incubated with 2.4 μg of SENP8 or 2 μg of USP2 at 37°C for two hours. All reactions were terminated by incubating with 4X SDS-PAGE sample buffer at 100°C for 10 min. Samples were separated on 16.6% SDS-PAGE and further analyzed using western blotting with the anti-(His)_x6_ tag antibody. N8 indicates CrNEDD8 in the figure.
