# Supplementary material for: The Molecular Determinants of NEDD8 Specific Recognition by Human SENP8
Source: PLoS One. 2011 Nov 14;6(11):e27742. doi: 10.1371/journal.pone.0027742 (PMC3215745; doi:10.1371/journal.pone.0027742)
Supplement: Figure S2 — Sequence alignment of NEDD8 proteins from different plant organisms. Residues of position 51 and position 72 are indicated in square boxes respectively. As noted, ubiquitin contains a negatively charged residue at position 51, whereas NEDD8 contains a corresponding non-polar residue. Position 72 in ubiquitin is an arginine but not the corresponding alanine residue in NEDD8. Cr, Chlamydomonas reinhardtii; Vc, Volvox carteri; Ps, Picea sitchensis; Pp, Physcomitrella patens; Os, Oryza sativa; Sb, Sorghum bicolor; At, Arabidopsis thaliana; Da, Deschampsia antarctica; Zm, Zea mays; Gm, Glycine max; Vv, Vitis vinifera; Rc, Ricinus communis; Pt, Populus trichocarpa. (DOC) [file pone.0027742.s002.doc]

**SUPPORTING INFORMATION**

**Figure S2 Sequence alignment of NEDD8 proteins from different plant organisms.** Residues of position 51 and position 72 are indicated in square boxes respectively. As noted, ubiquitin contains a negatively charged residue at position 51, whereas NEDD8 contains a corresponding non-polar residue. Position 72 in ubiquitin is an arginine but not the corresponding alanine residue in NEDD8. Cr, *Chlamydomonas reinhardtii*; Vc,

*Volvox carteri*; Ps,

*Picea sitchensis*;

Pp, *Physcomitrella patens*;

Os, *Oryza sativa*; Sb,

*Sorghum bicolor*;

At, *Arabidopsis thaliana*;

Da, *Deschampsia antarctica*;

Zm, *Zea mays*;

Gm, *Glycine max*;

Vv, *Vitis vinifera*;

Rc, *Ricinus communis*;

Pt, *Populus trichocarpa*.
