# Supplementary material for: The Molecular Determinants of NEDD8 Specific Recognition by Human SENP8
Source: PLoS One. 2011 Nov 14;6(11):e27742. doi: 10.1371/journal.pone.0027742 (PMC3215745; doi:10.1371/journal.pone.0027742)
Supplement: Table S1 — The list of the primers used for the mutagenesis in the study. All CrRUB1 mutants were generated by PCR-based QuikChangeTM Site-Directed Mutagenesis Kit (Stratagene, La Jolla, CA), according to the manufacturer's instruction. The primers used for the mutagenesis were listed in the table. N8 indicates CrNEDD8. (DOC) [file pone.0027742.s003.doc]

**SUPPORTING INFORMATION**

**Table S1 The list of the primers used for the mutagenesis in the study.** All CrRUB1 mutants were generated by PCR-based QuikChange^TM^ Site-Directed Mutagenesis Kit (Stratagene, La Jolla, CA), according to the manufacturer’s instruction. The primers used for the mutagenesis were listed in the table. N8 indicates CrNEDD8.

| Mutant | Direction | Primer |
| --- | --- | --- |
| CrRUB1_Ub(E51N) | Forward | 5’-TCGCGGGCAAGCAGCTGAACGACGGCCGCACCCT-3’ |
|  | Reverse | 5’-AGGGTGCGGCCGTCGTTCAGCTGCTTGCCCGCGA-3’ |
| CrRUB1_Ub(R72A) | Forward | 5’-CCTGCACCTGGTGCTGGCCCTGCGTGGTGGTATG-3’ |
|  | Reverse | 5’-CATACCACCACGCAGGGCCAGCACCAGGTGCAGG-3’ |
| CrRUB1_Ub(E51N/R72A) | Forward | 5’-TCGCGGGCAAGCAGCTGAACGACGGCCGCACCCT-3’ |
|  | Reverse | 5’-CATACCACCACGCAGGGCCAGCACCAGGTGCAGG-3’ |
| CrRUB1_Ub(G53D) | Forward | 5’-AGCAGCTGGAGGACGACCGCACCCTGGCCGAC-3’ |
|  | Reverse | 5’-GTCGGCCAGGGTGCGGTCGTCCTCCAGCTGCT-3’ |
| CrRUB1_N8(N51E) | Forward | 5’-TTGCGGGCAAGCAGATGGAAGACGACAAGCAGGCC-3’ |
|  | Reverse | 5’-GGCCTGCTTGTCGTCTTCCATCTGCTTGCCCGCAA-3’ |
| CrRUB1_N8(A72R) | Forward | 5’-GCGGATCCATGCAGATTTTCGTCAAGAC-3’ |
|  | Reverse | 5’-GCCTCGAGCCCGCCACGCAGGCGCAGCA-3’ |
| CrRUB1_N8(N51E/A72R) | Forward | 5’-TTGCGGGCAAGCAGATGGAAGACGACAAGCAGGCC-3’ |
|  | Reverse | 5’-GCCTCGAGCCCGCCACGCAGGCGCAGCA-3’ |
| CrRUB1_N8(D53G) | Forward | 5’-AGCAGATGAACGACGGCAAGCAGGCCAAGGAC-3’ |
|  | Reverse | 5’-GTCCTTGGCCTGCTTGCCGTCGTTCATCTGCT-3’ |
